# Multi-level intersectional stigma reduction intervention to increase HIV testing among men who have sex with men in Ghana: Protocol for a cluster randomized controlled trial

**DOI:** 10.1371/journal.pone.0259324

**Published:** 2021-11-29

**Authors:** LaRon E. Nelson, Laura Nyblade, Kwasi Torpey, Carmen H. Logie, Han-Zhu Qian, Adom Manu, Emma Gyamerah, Francis Boakye, Patrick Appiah, DeAnne Turner, Melissa Stockton, Gamji M. Abubakari, David Vlahov

**Affiliations:** 1 School of Nursing, Yale University, New Haven, CT, United States of America; 2 Center for Interdisciplinary Research on AIDS, School of Public Health, Yale University, New Haven, CT, United States of America; 3 Yale Institute for Global Health, School of Public Health, New Haven, CT, United States of America; 4 RTI International, Washington, DC, United States of America; 5 Department of Population, Family & Reproductive Health, School of Public Health, University of Ghana, Legon, Accra, Ghana; 6 Factor-Inwentash Faculty of Social Work, University of Toronto, Toronto, ON, Canada; 7 Department of Biostatistics, School of Public Health, Yale University, New Haven, CT, United States of America; 8 Educational Assessment & Research Center, Accra, Ghana; 9 Priorities on Rights & Sexual Health, Accra, Ghana; 10 Youth Alliance for Health & Rights, Kumasi, Ghana; 11 College of Nursing, University of South Florida, Tampa, FL, United States of America; 12 Department of Psychiatry, Columbia University Irving Medical Center, New York, NY, United States of America; Public Library of Science, UNITED KINGDOM

## Abstract

**Background:**

Men with have sex with men (MSM) in Africa face high levels of stigma due to elevated HIV exposure (actual or perceived), same-sex practices, and gender non-conformity. These stigmas are documented barriers to HIV prevention and treatment. Most stigma-reduction interventions have focused on single-level targets (e.g., health care facility level [HCF]) and addressed one type of stigma (e.g., HIV), without engaging the multiple intersecting stigmas that MSM encounter. Determining the feasibility and acceptability of multi-level intervention of reducing intersectional stigma and estimating its efficacy on increasing HIV testing are needed.

**Methods:**

We proposed a mixed method study among MSM in Ghana. First, we will develop the intervention protocol using the Convergence Framework, which combines three interventions that were previously implemented separately in Ghana for reducing stigma at the HCF-level, increasing HIV testing at the peer group-level, and increasing peer social support at the individual-level. Then, we will conduct a cluster randomized controlled trial with four pairs of HCFs matched on staff size. HCFs within each pair are randomized to the HCF-level stigma-reduction intervention or control arm. MSM (n = 216) will be randomized to receive the group-level and individual-level interventions or standard of care control arm. MSM will be assigned to receive HIV testing at one of the HCFs that match their study assignment (intervention or control facility). The frequency of HIV testing between MSM in the study arms at 3 and 6 months will be compared, and the predictors of HIV testing uptake at the HCF, peer group and individual-levels will be assessed using multi-level regression models.

**Discussion:**

These findings from this study will provide important evidence to inform a hybrid implementation-effectiveness trial of a public health intervention strategy for increasing HIV case detection among key populations in sub-Saharan African communities. Accurate information on HIV prevalence can facilitate epidemic control through more precise deployment of public health measures aimed at HIV treatment and viral load suppression, which eliminates risk of transmission.

**Trial registration:**

This study was prospectively registered on ClinicalTrials.gov, Identifier: NCT04108078, on September 27, 2019.

## Introduction

In Ghana, men who have sex with men (MSM) are at high risk of HIV acquisition and have low HIV testing rates, often delayed until the point of illness. HIV prevalence among MSM is over eight times higher (18%) than in the general population (2%) [1–4] and higher among MSM in some communities, such as Accra (34%) and Kumasi (14%) [5]. Data on HIV testing among MSM is limited. A 2011 study found two-thirds of MSM in Accra and Kumasi had not been tested for HIV in the past 12 months [3], while a 2012 study found that 40% of MSM respondents had never tested for HIV [6]. Moreover, a qualitative study found infrequent HIV testing practices among MSM due to stigma and inaccurate information [7]; another reported delayed HIV diagnosis, evidenced by reports of having advanced HIV symptoms prior to testing [8, 9].

The intersection of distinct stigmas experienced by MSM in Ghana (e.g., HIV, same-sex and gender-non-conforming stigmas) produces a unique and synergistic effect on HIV testing behavior, hereafter called intersectional stigma. MSM face intersectional stigma which can be experienced, perceived, anticipated, and internalized. At the organizational (i.e., healthcare facility [HCF]) level, HIV stigma manifests globally in multiple forms ranging from verbal abuse to refusal to treat and avoidance behaviors like double gloving only with clients living with HIV; the latter driven by unwarranted fears of HIV transmission [10–13]. A 2017 HCFs survey in Ghana documented high levels of both drivers (fear of HIV transmission, stigmatizing attitudes) and manifestations of HIV stigma among medical and support staff [14, 15]. Same-sex and gender-non-conforming stigma in HCFs manifests in a range of forms from verbal harassment (e.g. harsh, intrusive, unnecessary questioning) to unwillingness to care [7]. Twenty-nine percent of surveyed HCF staff in Ghana indicated that if they had a choice, they would prefer not to provide services to MSM [15]. At the interpersonal level (i.e., MSM peer groups) men may experience, perceive or anticipate HIV, same-sex, and gender non-conforming stigma from their peers [16, 17]. These intersecting stigmas manifest through attitudes, gossip, verbal abuse and social isolation of peers who they associate with HIV (regardless of serostatus) and who express their gender identities in ways that do not conform to perceived cultural norms of masculinity (i.e., gender non-conforming) [18]. At the intrapersonal (individual) level, HIV, same-sex and gender non-conforming stigmas may be experienced [19], internalized [20], perceived [21], vicarious [22, 23], or anticipated [22, 23]. In Ghana, MSM have reported routinely experiencing HIV, same-sex and gender non-conforming stigma [9, 18]. Even HIV-negative MSM experience HIV stigma because of normative beliefs that HIV is divine retribution for same-sex behavior.

Intersectional stigma is a significant barrier to engagement in the HIV prevention and treatment cascade. Intersectional stigma prevents MSM from seeking HIV testing and can undermine motivation for engaging in HIV prevention practices. The presence of HIV stigma in Sub-Saharan Africa leads to guarded disclosures which are disruptive to HIV testing and early linkage to care [21, 24]. Qualitative studies in Ghana found same-sex stigma undermines HIV testing for MSM [7] and was a barrier to seeking general health and HIV testing services [25]. Within HCFs, negative interactions with staff can discourage MSM from seeking HIV testing or disrupt linkage to care [25]. At the interpersonal and individual levels, perceived stigma can lead MSM to avoid health care services due to fear that someone will discover they have sex with men [21]. Anticipated stigma can generate fear of potential discriminatory treatment at HCFs, which may lead MSM to avoid or delay accessing services [26, 27]. At the individual level, internalized stigma may undermine motivation for engagement in HIV prevention activities and services [20].

Individual and interpersonal factors, such as self-efficacy and social support, are key determinants of HIV testing and risk reduction among MSM. Building resilience and peer-based social assets is critical to reducing stigma and facilitating engagement in health promoting behaviors [28–33]. Although it is critical to have interventions that focus on reducing the prevalence and intensity of stigma in structural environments (e.g., HCF, workplace, religious institution) as a means of reducing MSM’s exposure to stigma; it is also important that MSM be able to build interpersonal-level (peer-group) and intrapersonal-level (individual) capacities to challenge stigma, recover from stigmatizing experiences and to use strategies to protect against internalization of stigma (self-stigma) that may result from chronic and episodic exposures to stigma [34, 35].

### Study rationale and aims

Stigma occurs at multiple socio-ecological levels; therefore stigma-reduction interventions must also target multi-level sources of stigma. Although there are multi-level HIV prevention interventions targeting MSM [36–38], most stigma-reduction interventions occur at a single socio-ecological level or address only one stigma [39, 40]. The need and importance of tackling stigma at multiple levels in Ghana is highlighted as a priority in Ghana’s National HIV and AIDS Strategic Plan 2016–2020 [41] and the Ghana National HIV and AIDS Anti-Stigma and Discrimination Strategy for 2016–2020 [42]. Single-level interventions, while important, have limitations in fully addressing intersectional stigma to improve HIV testing outcomes, given stigma occurs and influences HIV testing at multiple socio-ecological levels. To develop and evaluate multi-component and multi-level interventions to maximize the efficacy, our study aims include Aim 1: To evaluate the feasibility and acceptability of a multi-level intervention to address intersectional stigma; Aim 2: To collect preliminary data for estimating the effect size of the intervention for scale up to a definitive efficacy trial.

## Methods

### Study design

**Fig 1:**
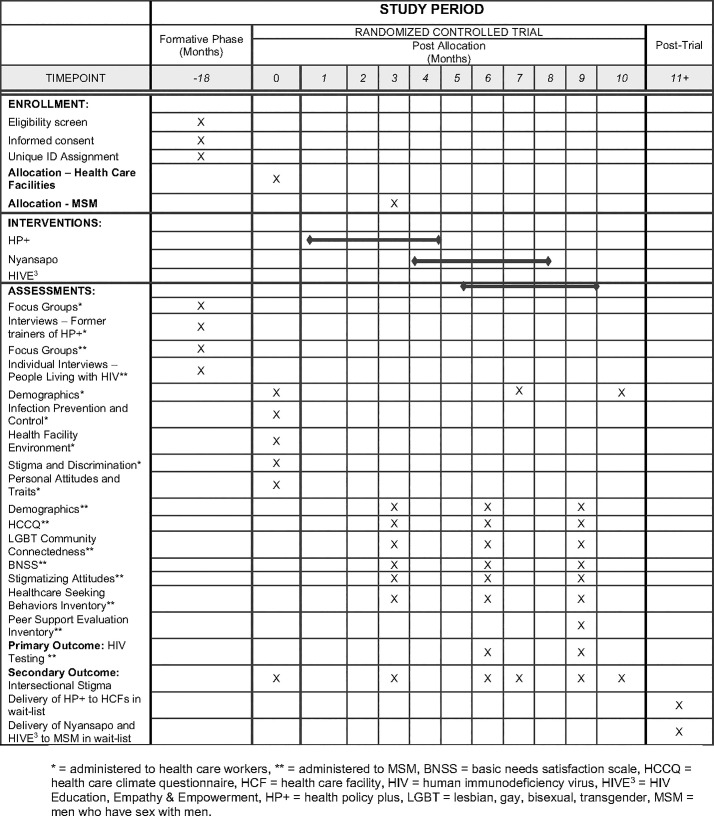
Schedule of enrollment, interventions, and assessments. * = administered to health care workers, ** = administered to MSM, BNSS = basic needs satisfaction scale, HCCQ = health care climate questionnaire, HCF = health care facility, HIV = human immunodeficiency virus, HIVE 3 = HIV Education, Empathy & Empowerment, HP+ = health policy plus, LGBT = lesbian, gay, bisexual, transgender, MSM = men who have sex with men.

**Fig 2:**
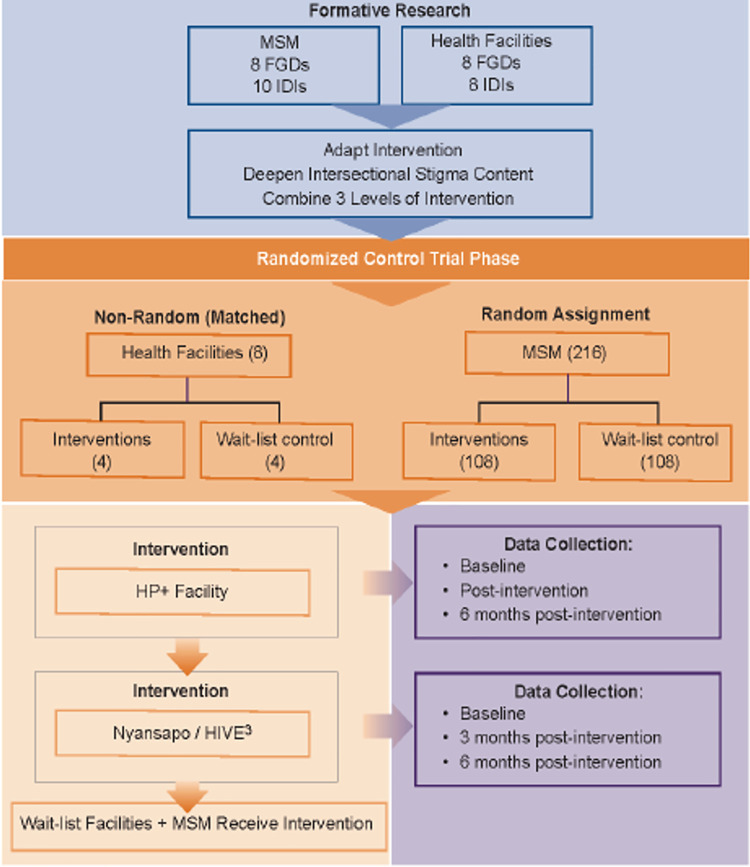
Diagram of study design for formative and randomized controlled trial phases.

The description of the study protocol conforms to the specifications of the Standard Protocol Items Recommendations for Intervention Trials (SPIRIT) checklist displayed in Fig 1. This study consists of a formative phase and a trial phase (see Fig 2). In the formative phase, we will use qualitative methods to identify the drivers and manifestations of intersectional stigma in HCFs and among MSM. These data will be used to adapt the existing interventions to enhance their focus on intersectional stigma-reduction and to combine the three interventions by interweaving select content and activities. In the trial phase we will assess the acceptability of the interventions, the feasibility of the cluster-RCT and estimate intervention effect sizes on HIV testing among MSM.

### Study setting

The study will be conducted in eight communities, four each in the Ashanti and Greater Accra regions of Ghana. Accra and Kumasi, the regional capitols, have the highest proportions of MSM living with HIV in the entire country. Furthermore, HIV testing and access to services remain low among MSM in these communities.

### Study participants

#### HCF staff

The study sample will consist of staff from HCFs in Ghana (n = 290; 90 in formative phase; 200 in RCT phase). We expect that the HCF staff sample will be predominantly composed of cisgender women (75%) based on our previous work in HCFs which show that women represent the majority of HCF staff. Although the health facility stigma-reduction intervention will be provided to a minimum of 70% of HCF staff in each facility, we will collect baseline and follow-up data on a random sample of 200 HCF staff in the RCT phase.

#### MSM

The study sample will also consist of Ghanaian MSM (N = 256; 40 in formative phase, 216 in RCT phase) ages 18 years and older. The MSM sample will be drawn from the Greater Accra and Kumasi metropolitan areas. We expect considerable ethnic and cultural diversity in the sample. The study is limited to cisgender MSM; however, we expect that >99% of the men screened will be cisgender.

### Eligibility

#### HCF staff

For the intervention, HCF staff are eligible to participate in the study if they are employed at one of the eight facilities identified for this study and identified with a potential to encounter MSM clients. HCF staff will be excluded if they anticipate leaving work at the HCF during the study period. For the FGDs, HCF staff are eligible to participate in the FGDs if they are employed at a study participating facility. HCF staff who have been previously trained in the HCF HIV stigma-reduction intervention that is being adapted for this study and have previously delivered it in a Ghanaian HCF are eligible for in-depth interviews.

#### MSM

For the intervention, MSM participants must be 18 years or older, assigned male sex at birth, currently self-identify as a cisgender man, report sexual activity with another man at least once within the previous six months and report that they are HIV-negative or status unknown. MSM will be excluded if they report plans to move out of the area during the study period. For the FGDs, MSM are eligible if they are 18 years or older, assigned male sex at birth, currently self-identified as a cisgender man, and reported sex with another man at least once within the previous six months. HIV status is not an inclusion criterion for MSM FGD participation. To participate in the IDIs, MSM must meet the same inclusion requirement for FGD participants, in addition to self-reporting that they are a person living with HIV.

### Ethics approval

This study was approved by the institutional review board of four organizations: Yale University (United States) IRB on October 9, 2019: 2000025917 Noguchi Memorial Institute for Medical Research (Ghana) IRB on November 6, 2019: CPN 016/19-20 Ghana Health Service Ethics Review (Ghana) on November 29, 2019: GHS-ERC 014/10/19 University of Toronto (Canada) on January 30, 2020: 00038738.

### Description of multi-level intervention

Guided by the Convergence Framework for selecting and combining interventions across levels we will combine three previously implemented (in Ghana) single-level interventions to address the multiple-levels of intersectional stigma faced by MSM, at the: HCF- (HP+),[15] interpersonal group- (Nyansapo) [43, 44] and intrapersonal-levels (HIVE^3^]) [45]. A summary of the three single-level interventions is presented in Table 1.

**Table 1:** Summary of the Three Single-Level Interventions for the proposed study.

Intervention	Level	Modality	Sessions	Length (Mins)
Health Policy Plus (HP+)-Total Facility approach	Health Facility	Participatory Group Training using: *Discussion Small group* *work* *Buzz groups Card storms Case Studies Role Plays/* *Drama Rotational* *brainstorms Picture tools*	1. Naming stigma through pictures 2. Stigma reflection 3. Confidentiality and stigma 4. Analyzing stigma in health facilities 5. Fears about non-sexual HIV transmission/ quantity, quality, and route of entry 6. Overcoming fear with standard precautions 7. Breaking the sex ice 8. Gender and sexual diversity (GSD) concepts 9. GSD terminology 10. How stigma impacts human rights 11. Panel discussion with people living with HIV 12. Challenge the stigma—be the change! 13. Writing a code of practice and action plan	60
				75
				60
				45
				60
				45
				20
				60
				30
				60
				60
				45
				120
Many Men, Many	Peer-	Group Sessions using: *Brainstorms Role Play* *Small group activity Paired work Develop menu* *of prevention options* *Action planning*	1. Ghanaian MSM and dual identity 2. HIV/STD prevention for Ghanaian MSM: the roles and risks for tops and bottoms 3. HIV/STD risk assessment and prevention options 4. Intentions to act and capacity for change 5. Relationship issues: Partner selection, communication, and negotiation of roles for Ghanaian MSM 6. Social support and problem solving to maintain change 7. Building bridges and community	140
Voices /3MV	Group			210
adaptation for Ghana-				
***Nyansapo***				110
				170
				140
				125
				130
HIV Empathy,	Individual	One-on-one	Peer mentors reach out to MSM participants for	varies
Education &		phone-based	informal discussion on affirming identities,	
Empowerment/HIVE^3^		contact, text or voice	information regarding HIV prevention and testing and offering emotional support	

#### The health policy plus (HP+) total facility stigma-reduction intervention (organizational/HCF-level)

The HP+ stigma-reduction curriculum includes 10–14 participatory exercises delivered over a two-day period to groups of 30 staff mixed by department and level (clinical/non-clinical) to ensure facility services are not disrupted, as well as to build joint understanding and motivation for stigma-reduction [46]. In addition, champion groups emerging from the training at each facility developed and implemented additional stigma-reduction interventions. The HP+ Ghana pilot evaluation study trained 1,228 HCF staff of all levels (clinical/non-clinical) in five facilities over a span of 4 months, averaging 79% coverage of staff across facilities [47]. This was possible because of the approach, which trained health facility staff and clients living with HIV or from other stigmatized groups (e.g., MSM, sex workers) from each of the intervention facilities to deliver the two-day participatory stigma-reduction training in their facilities. For this study, all eight selected facilities will provide detailed lists of their staff, by staff category, indicating their interest and willingness to participate. The HP+ intervention, which primarily addressed HIV stigma, will be refined to deepen and expand the focus on intersectional stigma, including updating the current training manual. The facilities that are assigned to the comparison condition will be offered the opportunity to receive the HP+ intervention after the RCT phase of the study is completed.

#### Nyansapo intervention (interpersonal/peer-group level)

Nyansapo is a Ghanaian adaptation *of Many Men Many Voices* [43, 48], a group-level behavioral intervention with demonstrated efficacy in increasing odds of HIV testing and reducing in Black MSM in the United States. *Many Men Many Voices* is designated as a best-evidence intervention by the Centers for Disease Control and Prevention (CDC) and is included in the CDC’s compendium of effective HIV prevention interventions [44]. Nyansapo previously demonstrated feasibility and acceptability during pilot program implementation in a community-based organization in Ghana [49]. MSM who are assigned to the intervention condition will be given dates for the weekend intervention retreats they would attend at temporarily rented facilities. Prior to attending Nyansapo, participants will attend an orientation meeting at the offices of local partner organizations to obtain information about the logistics and format of the retreat and to establish ground rules for appropriate behavior. Once participants arrive at the retreat, the Nyansapo intervention will be delivered in six consecutive 2- to 3- hour sessions. The Nyansapo intervention manual will be further refined to focus on intersectional stigma. Individuals assigned to the comparison condition will be offered the chance to participate in Nyansapo after the RCT is completed.

#### HIVE^3^ intervention (intrapersonal/individual-level)

HIVE^3^ provides three forms of support that are designed to counteract stigma: informational, emotional, and affirmation. Mentors provide emotional and affirmational support through attentive listening, encouragement, reassurance, and caring reflection. Peers foster empathy, acceptance, comfort, validation and help restore self-esteem and motivate persistence in times where stigma has caused stress in a MSM’s life. Informational support is demonstrated in the form of factual input, suggestions, feedback or advice, troubleshooting problems, relevant resources, and alternative courses of action. Support is provided via test message through a secure app that participants can download to their phones. Peer support can also be provided verbally, via the app, using a voice-over-internet protocol which serves to protect the peer mentors and mentees’ personal phone numbers. Participants will be provided a link to download the app. The app can only be accessed via secure using login and password. Messages are stored in an encrypted cloud and not maintained locally on the mobile phone device. A peer is a person internal to the community, with similar demographics and specific knowledge from lived experience. The provision of standardized training to peer mentors is essential for them to practice skills and orient them to the HIVE^3^ program; however, the training is not design to “professionalize” the mentors. It important that the mentors maintain their roles as informal and accessible peers [50–52].

### Study measures

#### Qualitative measures

In-depth interviews and focus groups will be used to further understand intersectional stigma, barriers and access to care and prevention from the perspective of both HCF staff and MSM. Key concepts to be addressed include: attitudes related to PLHIV, MSM and gender non-conforming men, previous care, and experiences within the healthcare system.

#### Outcomes

For MSM, we will assess important baseline characteristics for describing the sample, including: age, income, gender, education, ethnicity, relationship status, religion, sexual identity, sexual practices, HIV knowledge, HIV testing readiness, HIV testing history and histories of experiencing intersecting stigmas, access and utilization of health and social services, civic engagement and neighborhood. For HCF staff, we will assess a more limited set of baseline characteristics. Our primary outcome will be an uptake of HIV testing within six months of randomization. We will also record the frequency of HIV testing within six months, but we anticipate that few participants will take more than 1 test in this short period. Our secondary outcome will be a reduction in intersectional stigma, both at the HCF-level and among MSM participants. For HCFs, we will construct a latent variable of intersectional stigma using items from questionnaires that measure HIV stigma and discrimination and LGBT stigma among health facility staff. For intersectional stigma among MSM, we will also create a latent variable that is drawn from three scales that measure HIV stigma, same-sex stigma, and gender non-conforming stigma. Outcomes will be assessed within 3-and 6-months post-intervention for HCFs and 3- and 6-months post-intervention for MSM.

### Study procedures

The eight-step ADAPT-ITT framework [53, 54] will guide our approach to enhancing the interventions’ content to address intersectional stigma. Aim 1 will encompass steps 1–7, leading to the combined multi-level intervention adapted to deepen intersectional stigma content and step 8 in which we will conduct a waitlist-controlled RCT of the adapted intervention for feasibility and acceptability. An overview of this process is depicted in Fig 2.

#### Step 1 (Assess)

FGDs and IDIs will be conducted with MSM and HCF staff to inform the adaptation of the three existing interventions to deepen intersectional stigma content and their integration with each other to address intersectional stigma at multiple levels. FGDs were chosen because of their demonstrated suitability in studies of sexual health [55–58] and to capitalize on the spontaneous conversational interaction that occurs in groups. This re-creation of the social dynamic with the HCF staff and MSM is critical to the understanding of intersectional stigma from the social organization of these two groups. We also conduct a limited set of IDIs to examine more in-depth, topics that elicit intimate personal accounts that may not be suitable for sharing in groups. For example, MSM living with HIV are targeted for IDIs to gain insight into whether and how intersectional stigma experiences influenced their prevention behaviors, access to prevention services, timing of HIV testing and linkage to care. We will qualitatively investigate (1) the drivers and manifestations of HIV, same-sex and gender non-conforming stigmas (intersectional stigma) within HCFs and MSM peer groups, (2) how these intersecting stigmas undermine HIV testing and (3) perspectives on strategies for reducing intersectional stigma within HCFs and among MSM peer groups. This information will guide how these stigmas are addressed in the intervention.

#### Step 2 (Decide)

Guided by the formative research results, the team will meet to discuss what specific intervention activities should be modified to address intersectional stigma. This will include a review of the current intervention activities and discussion of how they can be refined to address intersecting stigmas. In consultation with our civil society partners, we will incorporate these changes into the curriculum, identify gaps/errors in logic and make corrections in preparation to conduct a workshop of the intervention with key reference groups.

#### Step 3 (Administer)

After initial preliminary modifications, we will convene a group of programmatic, scientific and technical experts to review the proposed intersectional stigma modifications to the three interventions. The group will convene in a series of workshops conducted over a period of 4 days and will include individuals with experience in delivering each of the three interventions in their original forms, as well as selected individuals who will conduct the qualitative data collection and analyses in the formative phase. We will leverage online videoconference technology to conduct joint workshop sessions (i.e., stakeholders for HP+, Nyansapo and HIVE^3^) because it is consistent with the concept that the intervention must retain relevance to MSM at all points along the HIV testing pathway, including in the HCF and among their peer-groups. Additionally, we will use online breakout rooms to engage in small group discussions where participants can react to the preliminary modifications presented as well as generate ideas for how to improve the responsiveness of the intervention curricula to addressing intersectional stigma.

#### Step 4 (Produce)

Based on the feedback from the simulation workshop participants and our observations of their engagement with the activities, we will determine what content and approaches to incorporate into the adapted intervention manual and which content and processes to edit or forego. In our decision-making process, informed by the qualitative formative research results, we will give priority consideration to maintaining components that have the highest consistency with scientific literature on intersectional stigma, local cultural relevance, and likelihood to be fun/enjoyable-key requisites for successful intervention uptake.

#### Step 5 (Topic)

We will engage topic experts to review the adapted manual and provide feedback on its congruence with the original intervention and local sociocultural relevance. A copy of each adapted intervention manual will be provided to the principal investigators of the original separate level interventions and be reviewed by experts in intersectional stigma. Partnering organizations will give feedback that is key to the local cultural relevance and intervention implementation.

#### Steps 6 (Integrate) and 7 (Train)

In this step, we will take feedback provided by the topic experts and use it to make final revisions to the intervention manual. We will also produce updated training manuals to standardize training and permit future replication of the intervention in other settings. We will provide comprehensive training to study staff whose roles are dedicated to delivering the intervention. We will develop the training to be multi-faceted to facilitate comprehension and retention of training concepts, using strategies successfully deployed in other HIV prevention research projects.

#### Step 8 (Test)

We will conduct an RCT of the multi-level intervention with a 6-month wait-list control. In each city, we will create two pairs from the 4 HCF sites matched by the number of HIV tests performed in the calendar year 2020. We will randomly assign clinics (enveloped odd/even) within each pair to either receive the HP+ or the wait-list control. MSM will be randomly assigned to receive either Nyansapo and HIVE^3^ or wait-list control. MSM in the intervention group will be asked to follow-up for HIV testing and services at one of the HCFs assigned to the HP+ intervention and the MSM in the wait-list control group will be asked to follow-up at any of the HCFs in wait-list control. Nurses from the intervention HCFs will participate as guest facilitators in one of the Nyansapo sessions and will use that opportunity to reach out to MSM to visit their facility for testing. Our primary hypothesis is that the multi-level intervention will increase uptake of HIV testing among MSM participants. We will use research data collectors to measure frequency of HIV testing among MSM at 3- and 6-months post-intervention using a combination of self-report survey and verification by study nurses (for men who get tested for HIV at participating HCFs.) Our secondary hypothesis is that the multi-level intervention will reduce intersectional stigma that HCF staff convey and that MSM experience in HCFs. We will use research data collectors to administer a survey measuring intersectional stigma 1) in the HCFs at baseline, 3-months post and 6-months post intervention and 2) among MSM at baseline, 3- and 6-months post intervention.

### Sample size

Sample size calculation for MSM participants is based on testing the primary study hypothesis that multi-level intervention will increase the HIV testing rate. Our previous study showed that the 6-month HIV testing rate among MSM was 40%. We hypothesize that this rate will increase to 70% after six months of multi-level intervention. This estimate is based on the evidence from the 3MV trial which showed 81% greater odds of HIV testing in intervention compared to the control group at 6-months and is conservative in comparison to UNAIDS “90-90-90” goals. To determine whether our estimated rate of 70% following interventions is different than the comparison rate of 40% with 80% power and Type I error probability 0.05, while assuming a 10% intra-cluster correlation coefficient, we need 86 MSM participants in each arm, or 172 in two arms by the end of 6-month follow-up. Given a cohort retention rate of 80% (based on retention in previous studies with MSM in Ghana), a total of 216 MSM will be recruited. To evaluate the impact of intervention training on intersectional stigma among staff, we will test whether the training intervention will increase the willingness of HCF staff to provide services to MSM. The historical data shows that the willingness rate is 75%. We expect that the intervention will increase the willingness rate up to at least 90% in the intervention clinic staff, while there is no change in the control group, and we will need 97 staff in each arm, or 194 in total, to achieve 80% power with a 5% Type I error rate.

### Recruitment

This study involves two phases (formative phase and randomized controlled trial phase) and two target population groups: HCF staff and MSM. There are specific criteria for recruitment for each population group in each of the study phases. The investigative team is partnered with three Ghanaian civil society organizations who will implement the research activities in their respective geographic regions and target populations. Two are MSM-led organizations that have worked with the team on previous studies. Both organizations have a track record in recruitment and retention of MSM in research studies. The third organization is a Ghanaian NGO that provides research and intervention implementation technical assistance services in Ghana, including for stigma-reduction intervention research, and brings strong relationships with the health sector in Ghana.

#### HCF staff

Recruitment for the intervention will target training a minimum of 70% of HCF staff who will encounter MSM seeking HIV testing at the facility (e.g., nurses, lab staff, guards, receptionists) and key staff (influencers) from different cadres of staff in other departments where MSM may need to seek health services (e.g., outpatient department, men’s in-patient ward, genitourinary, emergency). We will recruit staff that represent a diverse cross-section of the facility who MSM are likely to encounter during the process of HIV testing (e.g., receptionist, security guard, nurse, HIV counselor, cashier). We will secure an updated list of HCF staff (HCFs already provided a list of staff by category in support of the proposal) and construct a random sample that will be contacted from the list, while allowing for staff to confidentially decline participation. For the formative research, each FGD will include 6–8 persons stratified by medical versus non-medical staff. We will also conduct IDIs with HP+ HCF staff and client trainers (n = 10) who were trained as facilitators under our previous work and who have been delivering the HCF HP+ intervention to HCFs in Ghana not involved in our proposed study.

#### MSM

We will recruit MSM using Starfish sampling—a combination or venue-based/time location sampling and peer-referral sampling strategies [59]. We have used both recruitment strategies separately in previous studies; however, recent evidence demonstrates that the Starfish Sampling (combining the two strategies) optimizes the recruitment of yield of populations that are considered hidden. We will conduct face-to-face recruitment through our civil-society partners serving MSM in-house programs and outreach activities. We will also recruit using coupon-based peer-referrals, which are a component of respondent driven sampling (RDS) [60–62]; however, our interest is in recruiting a sufficient sample size of MSM; therefore, we will not apply statistical weights to attempt to generate a population estimate that is the hallmark of the RDS approach. We established region-specific enrollment targets [Accra (n = 108), Ashanti (n = 108)] based on estimation of MSM population sizes in each city. We will employ and train research assistants from outreach staff at our two civil society organizations who already have extensive experience engaging MSM for research and program activities.

### Statistical analysis plan

The intervention outcomes will be assessed using a rigorous intention-to-treat approach where participants are included in the analysis as assigned initially, regardless of whether they receive the multi-level intervention or usual care. First, we will compare baseline data to see if randomization made the groups equivalent. If we determine non-equivalence, then the non-equivalent variables will be accounted for in the final analyses using a difference in differences analytic technique where randomization is used to minimize any perception of bias in the selection of sites by investigators. We will determine the proportions of HIV testing at 3- and 6-month follow-up assessments among MSM with 95% confidence intervals (CI). To test the primary study hypothesis that multi-level intervention will increase the uptake of HIV testing within six months of randomization, we will use an uncorrected chi-square statistic.

Additionally, multivariable logistic regression will be used to compute the adjusted odds ratio and 95% CI for the binary outcome–HIV testing. The successful uptake of HIV testing can be dependent on the environment in the HCF so that HIV testing among MSM participants at the same HCF may be corrected or “clustered.” To account for the potential post-randomization clustering effect in this individually randomized clinical trial, a generalized linear mixed-effects model with a logit link function will be fitted, and this model includes both HCF cluster-level and MSM individual-level factors. Structural equation modeling will be performed to evaluate the association between the multilevel intervention and uptake of HIV testing while simultaneously assessing the mediation role of intersectional stigma. We will also perform the same analyses for HIV testing within three months of randomization, although the analyses may be underpowered. The likelihood of intervention contamination across the study groups could not be excluded, because some participants may not receive the respective interventions to which they are randomized, e.g., some members of the control group may seek health care at the intervention HCFs during this trial. Therefore, we will also perform per-protocol analysis, which includes only those participants who strictly adhere to the protocol and report the results along with the results of the intention-to-treat analyses. To estimate the effect size of the HP+ intervention for staff, we will compare the rates of willingness to provide healthcare to MSM between the staff in intervention and control HCFs. Chi-square test and t-test will be used for the binary (willingness) and continuous (stigma score) outcomes, respectively. Multivariate logistic or linear regression models will be fitted while adjusting for confounding factors such as sex and job title of the staff.

### Ethical considerations

#### Process of consent/Assent

The research assistants will be responsible for obtaining and ensuring informed consent by study participants. In each study phase, the research assistant will fully explain the study to potential participants and answer all questions regarding what participants will be asked to do as part of the study. Research assistants will receive training on informed consent so that during any community-based outreach activities, they can tell potential participants what to expect. In most cases the research assistant will perform the informed consent procedures as a contiguous process with recruitment. We will ensure that all participants know that their participation is completely voluntary and that they can withdraw at any time, without repercussion. Once a participant verbally indicates to the research assistant that all their questions have been satisfactorily answered, we will document that she or he has given informed consent to participate by having the participant sign an informed consent form. For HCF staff, we will only document informed consent for data collection. Consent for the HP+ intervention will be obtained at the HCF-facility level (not the staff-level). HCF staff may participate if, and only if, they want to participate in the intervention. All participants will be reminded that neither participating nor declining to participate has any impact on their employment status (HCF staff) or service provision (MSM).

We will document that she or he has given written informed consent to participate by having the participant sign an informed consent form (paper). As stated above, For HCF staff, we will only document informed consent for data collection. We understand that the informed consent does not end when it is documented but continues throughout the entire time that the participant is engaged with the study. In service of this critical point, the research assistants are responsible to ensure that the participant understands what she or he is being asked to do as part of the study throughout the time that the individual is enrolled so that is participation remains informed and volitional at all times. This will be assessed by changes in cognitive functioning or decisional impairment and assessed as needed by study staff.

We will use electronic forms to document written informed consent for MSM. This is an important security measure that avoids having a participant’s name affixed to a paper document that describes the study. Same-gender sexual behavior is still largely stigmatized in Ghana and the inadvertent disclosure of this may lead to social harms. The use of electronic documentation (e.g., signing the name into a computer tablet based touch-screen) is logistically less complex by reducing the handling of paper and it minimizes the risk of privacy breach by storing the information on a secure password-protected cloud platform—not on the computer tablet device itself.

#### Confidentiality and security of data

We will only collect the minimum contact information necessary to be able to reach participants for scheduling and reminders about the study visits, HP+ intervention, 3MV/Nyansapo intervention, and HIVE^3^ peer support sessions and data collection. We will collect a participant’s first name (or alias), cell phone number, and e-mail addresses. We will not collect information such as home address as to avoid the risk that unauthorized persons can apprehend and use it to identify study participants. We will also ask participants to provide an emergency number of a trusted family member or friend that we can call in the event of a medical or legal emergency. For HCF staff, we will also record the HCF site where they are employed. No research materials will contain the participant’s name. Instead, Participant ID numbers will represent a participant’s identity and will be the only link to identifying information. This key, linking participant IDs and contact information, will be stored alone on an encrypted server. At study completion, the key will be destroyed.

Three sites will be responsible for data collection and intervention delivery. The Ghanaian research NGO will provide data collection and data management services for the overall study. This will include establishing and training a team of research assistants who will obtain informed consent and conduct data collection in the formative and randomized controlled trials phases. They will also oversee data collection and intervention implementation in the health facilities. We will recommend that participants complete the informed consent and survey procedures inside of the eight participating HCF sites. Participants will be given a paper version of the survey by study staff; study staff will then collect the surveys and enter the data into an electronic database. Data entry will be verified and the paper surveys will be destroyed. HCF staff surveys will be anonymous; HCF respondents will not be asked to provide their name, specific age, or other identifying information on the survey.

The two MSM-led organizations will provide recruitment, data collection and retention services for MSM in the study. Research assistants will recruit, obtain informed consent, collect data, and follow-up with participants to support study retention. The research assistant will record all client contact information in an online data management information system via computer tablets. We will recommend that participants complete the informed consent and survey procedures inside of our partner’s data collection sites. Survey procedures will include coming to a data collection site and completing the survey using a tablet. No paper data will be collected and data will be automatically stored in the online data collection system. If a participant prefers, a study staff member can read the questions to the participant and document the participant’s answers.

Permission to collect HIV testing data will be requested in the informed consent form. We will also ask for participants to complete an authorization for medical records release. For those who report a post- intervention HIV test, we will seek to get confirmation from the HCF that test was conducted and the test result. For participants who report HIV testing at their assigned HCF, the study nurse will verify study participants’ name against the HCFs HIV test log to confirm the test was done at the facility. The study nurse will use an internet enabled computer tablet device to record the date of the test and test result directly into the online data management system used in the study.

We will destroy all identifiers after the data analyses have been completed. We define completed as the publication of the results of the primary research questions in a scientific peer-reviewed journal. Before that time, there may arise a need to audit the data to a level that is identifiable. We will maintain the capacity to conduct such a detailed audit until the primary results are published. To maximize participant confidentiality, all data will be identified only by the subject ID number, and no participant names will appear on any research materials. All research data will be maintained on a secure computer system with actively maintained high level of security to ensure the confidentiality of our databases. Access to the list containing participant IDs will only be provided on a need-to-know basis to approved study staff.

#### Data storage and access

Paper documents (HCF surveys) will be entered into an electronic database, verified, and destroyed immediately upon verification. The link between the participant ID number and identifiable information will be limited to approved study personnel on a “need to know” basis. The file will be password protected and stored on an encrypted server in a location with no survey data. This link will be kept until study completion, at which point it will be destroyed by the site PI. Additionally, all data from the study (whether identifiable or not) will be stored on an encrypted server, in a password protected folder.

To limit possible loss of confidentiality with the audio files, all qualitative data (HCF staff and MSM; FGDs and IDIs) will be protected using the following measures: 1) audio files will be internally transcribed as soon as possible, but within no more than 14 days after the interview date and all identifying information will be removed; 2) a second staff member will listen to the transcripts to verify they have been correctly transcribed and ensure no remaining identifying information is present; and 3) audio files will be permanently destroyed.

No one outside of the MPIs, research assistant and study medical director and healthcare providers with a clinical “need to know” will have access to any identifiable data. The research assistant will need access to basic identifying data such as name, phone number for follow-up contact purposes to aid in recruitment, enrollment and data collection. If an urgent clinical issue arises (such as, a participant experiencing acute psychological distress) it may be necessary to link the participant’s contact information to subject’s identity to provide clinical follow-up. No data will be shared without the participant’s written informed consent to release medical information. It is in keeping with the principle of beneficence to ensure continuity of care by making it possible (not mandatory) for participants to share information with a medical provider.

All data will be identified only by the subject ID number, and no participant names will appear on any research materials. MSM participants will be followed longitudinally and a minimal amount of contact information (phone, email, first name or alias) will be linked to the data by use of a corresponding Participant ID. The link between the participant ID number and identifiable information will be limited to approved study personnel on a “need to know” basis. The file will be password protected and stored on an encrypted server in a location with no survey data. This link will be kept until study completion, at which point it will be destroyed. Participation in the study does not involve more than minimal risk.

#### Data and safety monitoring plan

This study is minimal risk. The MPIs are responsible for monitoring the data, ensuring protocol compliance, and conducting the safety reviews monthly. During the review process the MPIs will evaluate whether the study should continue unchanged, require modification/amendment, or close to enrollment. The MPIs, the Institutional Review Boards (IRBs) or Noguchi Memorial Institute and Ghana Health Services have the authority to stop or suspend the study or require modifications.

#### Reporting of adverse events

This protocol presents minimal risks to the subjects and *Unanticipated Problems Involving Risks to Subjects or Others*, including adverse events, are not anticipated. In the unlikely event that such events occur, Reportable Events (which are events that are serious or life-threatening and unanticipated (or anticipated but occurring with a greater frequency than expected) and possibly, probably, or definitely related) or Unanticipated Problems Involving Risks to Subjects or Others that may require a temporary or permanent interruption of study activities will be reported immediately (if possible), followed by a written report within 5 calendar days of a Principal Investigator (PI) becoming aware of the event to the IRB (using the appropriate forms from the website) and any appropriate funding and regulatory agencies. The investigator will apprise fellow investigators and study personnel of all *Unanticipated Problems Involving Risks to Subjects or Others* and adverse events that occur during the conduct of this research project will be immediately reported to the MPIs and Site PI/Medical Director and will be discussed at monthly data and safety monitoring meetings.

The protocol’s Data and Safety Monitoring Board will be informed of any form of distress or adverse events within 5 days of the event becoming known to the PI; additionally, funding and regulatory agencies will be informed of serious adverse events within 5 days of the event becoming known to the principal investigator. All reports of distress will be immediately reported to the MPIs and Site PI/Medical Director and will be discussed at monthly data and safety monitoring meetings. All data regarding adverse experiences will be reviewed by the MPIs and the study medical director.

#### Management of interim results

This study will use a MPI approach due to the multisite, multi-level international nature of the study. Through the multi-PI approach, we aim to support NIH’s goal “to maximize the potential of team science efforts in order to be responsive to the challenges and opportunities of the 21st century”. The team organized for this study includes scientists from various disciplines including nursing, demography, public health, social work, epidemiology, and statistics. The MPIs will jointly provide oversight of the entire study including development and implementation of all policies, procedures and processes to ensure cultural relevance, congruence and sensitivity of the intervention modification and combination process; and protection of human subjects. In these roles, PI#1 and PI#2 will be responsible for the implementation of the Scientific Agenda, the Leadership Plan and the specific aims and ensure that systems are in place to guarantee institutional compliance with US laws, DHHS and NIH policies including biosafety, human subjects’ research, data and facilities.

#### Dissemination plan

Research resources generated with funds from this grant will be freely distributed, as available, to qualified academic investigators for non-commercial research. The study principal investigators will adhere to the NIH Grants Policy on Sharing of Unique Research Resources including the "Sharing of Biomedical Research Resources: Principles and Guidelines for Recipients of NIH Grants and Contracts" issued in December, 1999. https://grants.nih.gov/grants/intellproperty_64FR72090.pdf. Specifically, material transfers would be made with no more restrictive terms than in the Simple Letter Agreement or the UBMTA and without reach through requirements. Should any intellectual property arise which requires a patent, we would ensure that the technology remains widely available to the research community in accordance with the NIH Principles and Guidelines document. The study PIs will also take responsibility for the following activities in support of public dissemination: (1) register the awarded study with clinicaltrials.gov within 21 calendar days of the enrollment of the first participant, (2) submit the study’s final results to clinicaltrials.gov within 1 year of the study’s primary completion date, (3) disseminate the study results in peer-reviewed scientific journals, abstract presentations at scientific conferences that are appropriate to the content of the study and (4) develop a community-centered fact sheet and presentation on the study and its results and present it to local Ghanaian stakeholders, including Ghana AIDS Commission and the National AIDS Control Programme. In addition to the reporting the primary study outcomes, we intend to published peer reviewed scientific manuscripts the report on the study adaptation process, description of the multi-level intervention, and local community-participatory engagement.

#### Protocol modifications

In principle, the scientific leadership team will operate by consensus. The members of the study team have been jointly involved in the conception and planning of the proposed project and will continue to jointly share responsibility for making scientific decisions throughout the study period. If a question falls within one investigator’s direct area of expertise or experience (e.g., MSM community engagement, HCF facility engagement, statistical analysis), the other investigators will defer to that individual as the primary decision-maker. If a scientific question arises that the leadership team is unable to resolve by consensus, the investigator responsible for that component of data collection will have the authority to make the final decision.

#### Payments for participation

We will implement a modest, modular gratuity structure to be applied to participants who enroll and attempt to participate in the study. A cash gratuity will be offered for participation for each participant at baseline and follow-up study visits. These gratuities are culturally accepted gestures of appreciation for the participants’ generosity in contributing their time and knowledge to the study. Participants will receive gratuities immediately after completion of data collection. Participants will receive the gratuity for attempting to complete the activities at the study visits and can receive it even if they decide (at any point) that they do not wish to continue completing the study activities. If participants know that they will receive the gratuity even if they do not complete the survey, IDI, or FGD, then this will reduce the risk that participants are financially coerced to complete any component of the study.

## Discussion

This intervention utilizes a multi-level approach to increase HIV testing, and decrease intersectional stigma, experienced by MSM in Ghana. If the intervention is found to be successful, it has the potential to decrease rates of HIV among MSM in Ghana. This trial represents the next step toward this goal. In Aim 1 we will combine three interventions previously found to be efficacious. We will then conduct a waitlist-controlled RCT of the adapted intervention to assess feasibility and acceptability of the intervention. In Aim 2 we will estimate the effect size of the intervention on HIV testing and intersectional stigma reduction. At the end of this trial, we will have sufficient data to conduct a larger efficacy/effectiveness trial of the multi-level intervention.

This study has significant potential benefits to the scientific community and communities of healthcare workers and key populations, such as MSM. The study will generate knowledge that will be used to inform the development of intervention protocols that can help reduce intersectional stigma among HCF staff and MSM. These reductions in stigma can improve HIV testing and service engagement for prevention and treatment (including, but not limited to, HIV). Furthermore, this study will contribute to further development of the HIV implementation science program, which aims to make scientific advances through research while simultaneously making gains and improvements in HIV for key populations in real-world clinical practice environments.

## Supporting information

S1 Checklist SPIRIT 2013 checklist: Recommended items to address in a clinical trial protocol and related documents*. (DOC)

## List of abbreviations

3MV: Many Men Many Voices
ADAPT-ITT: Assessment, Decisions, Administration, Production, Topic Experts, Integration, Training and Testing Model
AIDS: Acquired immunodeficiency syndrome
CI: Confidence Intervals
DSMB: Data Safety and Monitoring Board
EARC: Educational Assessment Research Center
FGD: Focus Group discussions
GSD: Gender and sexual diversity
HCF: Health Care Facilities
HIV: Human immunodeficiency virus
HIVE^3^: HIV Empathy, Education & Empowerment
HP+: Health Policy Plus
IDI: In-depth Interview
MSM: Men Who have Sex with Men
MPIs: Multiple Principal Investigators
NIH: National Institute of Health–United States
PHI: Private Health Information
PLHIV: People Living with HIV
PI: Principal Investigator
PORSH: Priorities on Rights and Sexual Health
REDCap: Research Electronic Data Capture
RTI: Research Triangle Institute
RCT: Randomized Controlled Trial
RDS: Respondent-driven Sampling
STD: Sexually Transmitted Disease
UNAIDS: The Joint United Nations Programme on HIV and AIDS
YAHR: Youth Alliance for Health and Human Rights
